# Single center assessment of the role of Oakland score among patients admitted for acute lower gastrointestinal bleeding

**DOI:** 10.1186/s12876-024-03283-y

**Published:** 2024-07-15

**Authors:** Luis M. Nieto, Yihienew Bezabih, Sharon I. Narvaez, Chaturia Rouse, Charleigh Perry, Kenneth J. Vega, Jami Kinnucan

**Affiliations:** 1 Department of Internal Medicine, WellStar Cobb Medical Center, Austell, GA USA; 2 Division of Gastroenterology and Hepatology, Augusta University-Medical College of Georgia, Augusta, Georgia; 3 Division of Gastroenterology and Hepatology, Mayo Clinic, Jacksonville, FL USA

**Keywords:** Lower gastrointestinal bleeding, Oakland score, Baseline anemia, Hospital admission, Safety discharge

## Abstract

**Background/Objectives:**

The Oakland score was developed to predict safe discharge in patients who present to the emergency department with lower gastrointestinal bleeding (LGIB). In this study, we retrospectively evaluated if this score can be implemented to assess safe discharge (score ≤ 10) at WellStar Atlanta Medical Center (WAMC).

**Methods:**

A retrospective cohort study of 108 patients admitted at WAMC from January 1, 2020 to December 30, 2021 was performed. Patients with LGIB based on the ICD-10 codes were included. Oakland score was calculated using 7 variables (age, sex, previous LGIB, digital rectal exam, pulse, systolic blood pressure (SBP) and hemoglobin (Hgb)) for all patients at admission and discharge from the hospital. The total score ranges from 0 to 35 and a score of ≤ 10 is a cut-off that has been shown to predict safe discharge. Hgb and SBP are the main contributors to the score, where lower values correspond to a higher Oakland score. Descriptive and multivariate analysis was performed using SPSS 23 software.

**Results:**

A total of 108 patients met the inclusion criteria, 53 (49.1%) were female with racial distribution was as follows: 89 (82.4%) African Americans, 17 (15.7%) Caucasian, and 2 (1.9%) others. Colonoscopy was performed in 69.4% patients; and 61.1% patients required blood transfusion during hospitalization. Mean SBP records at admission and discharge were 129.0 (95% CI, 124.0-134.1) and 130.7 (95% CI,125.7-135.8), respectively. The majority (59.2%) of patients had baseline anemia and the mean Hgb values were 11.0 (95% CI, 10.5–11.5) g/dL at baseline prior to hospitalization, 8.8 (95% CI, 8.2–9.5) g/dL on arrival and 9.4 (95% CI, 9.0-9.7) g/dL at discharge from hospital. On admission, 100/108 (92.6%) of patients had an Oakland score of > 10 of which almost all patients (104/108 (96.2%)) continued to have persistent elevation of Oakland Score greater than 10 at discharge. Even though, the mean Oakland score improved from 21.7 (95% CI, 20.4–23.1) of the day of arrival to 20.3 (95% CI, 19.4–21.2) at discharge, only 4/108 (3.7%) of patients had an Oakland score of ≤ 10 at discharge. Despite this, only 9/108 (8.33%) required readmission for LGIB during a 1-year follow-up. We found that history of admission for previous LGIB was associated with readmission with adjusted odds ratio 4.42 (95% CI, 1.010-19.348, *p* = 0.048).

**Conclusions:**

In this study, nearly all patients who had Oakland score of > 10 at admission continued to have a score above 10 at discharge. If the Oakland Score was used as the sole criteria for discharge most patients would not have met discharge criteria. Interestingly, most of these patients did not require readmission despite an elevated Oakland score at time of discharge, indicating the Oakland score did not really predict safe discharge. A potential confounder was the Oakland score did not consider baseline anemia during calculation. A prospective study to evaluate a modified Oakland score that considers baseline anemia could add value in this patient population.

## Introduction

Gastrointestinal bleeding (GIB) is a frequent cause of hospitalization in the United States. Almost 4 million admissions with GI related-conditions occurred in 2018; GIB was the most common principal discharge code with more than half million of cases [1]. About 20% of those cases were caused by lower gastrointestinal bleeding (LGIB) and approximately 15% of the LGIB cases were readmitted within 30 days [1]. Initial assessment of a patient presenting with LGIB includes focused history, physical exam, and laboratory testing [2, 3]. Additionally, past medical history of LGIB on admission, medications that increase risk of bleeding, cardiovascular, cerebrovascular, hepatic and renal comorbidities should also be evaluated [3]. Assessment of these comorbidities allows for proper detection of high-risk patients and may alter management approach [3].

The Oakland score is a risk assessment score used to classify the severity of acute LGIB bleeding to predict safe discharge [2, 4]. To develop this score a multivariable logistic regression model was used to recognize predictors of safe discharge, then it was converted into a simplified risk scoring system [2]. Scores ≤ 8 indicate a 95% or higher probability of safe discharge. Probability of safe discharge is defined as absence of rebleeding, blood transfusion, therapeutic intervention, 28-day readmission or death [2, 5]. Scores > 8 indicate admission with further workup and resuscitation as necessary [2, 5]. External validation of the Oakland score revealed the threshold could be extended to > 10 for low-risk patients [5]. A 10-year retrospective study used to predict the severity of outcomes using Oakland score, determined a score ≥ 12 points were closely associated with adverse outcomes [6].

Patients presenting to the hospital with LGIB are less likely to require hospital admission, inpatient intervention, or experience an adverse outcome when compared with upper gastrointestinal bleeding cases, especially if those patients are identified as a low risk [5]. A valuable strength of the Oakland score is that it facilitates early clinical decisions with data commonly used in the emergency department without need for endoscopy data, then requiring hospital admission [4]. Oakland scores are useful in both early evaluation in the emergency department and predicting early aggressive management in hospitalized patients [6, 7]. In this study, we retrospectively evaluated if this score can be implemented to assess safe discharge (defined as a score ≤ 10) at WellStar Atlanta Medical Center (WAMC).

## Methods

### Study population and design

A retrospective cohort study using a single-institutional database from January 1, 2020, to December 30, 2021, was performed. All patients hospitalized at WellStar Atlanta Medical Center with lower GI bleeding during this period were eligible for the study. Data was extracted from electronic health record (EHR) including sociodemographic, clinical, laboratory and imaging findings as well as imaging was recorded using a standard data extraction protocol. Oakland scores was calculated for all patients at admission and discharge. We determined the association between readmission rates and Oakland score (and its components such as hemoglobin level at discharge). Also calculated was the Charlson Comorbidity index (CCI) as it could be a confounding factor for prolonged hospital stay. The primary goal of this assessment tool is to predict long-term mortality. The CCI’s initial version was modified to include a wide range of data sources, including ICD-10 codes which is used in our study. In numerous clinical groups, including the intensive care unit (ICU), where patients with LGIB are frequently admitted, CCI has been shown to predict long-term death [8].

### Inclusion and exclusion criteria

Patients admitted with a clinical diagnosis of lower GI bleeding during January 1, 2020, to December 30, 2021. Specifically, we included patients with ICD10 codes of K50.011, K50.111, K50.811, K50.911, K51.011, K51.211, K51.311, K51.411, K51.511, K51.811, K51.911, K55.21, K57.01, K57.11, K57.13, K57.21, K57.31, K57.33, K57.41, K57.51, K57.53, K57.81, K57.91, K57.93, K62.5, K62.6, K64.8, K64.9, K92.1, K92.2. Patients with upper GI bleeding defined by bleeding coming from a gastrointestinal source above the ligament of Treitz were excluded. For readmissions, we utilized the exact same inclusion criteria as for admissions.

### Study outcomes

The primary outcomes were: (1) mortality; (2) Assess the use of the Oakland score with discharge practices; (3) rates of colonoscopy; (4) 1-year readmission rate. Secondary outcome assessed was the impact of LGIB on resource utilization including: length of stay (LOS) and requirement of blood products in relation to Oakland score.

### Data collection

Patient demographics and clinical variables were collected from the EHR. Information on patient characteristics including age (years), gender and race. Principal diagnosis, inpatient complications, comorbidities, and procedures were also recorded. We also collected data on anemia history. We used the CCI to assess the burden of comorbid conditions.

### Statistical analysis

**Fig. 1:**
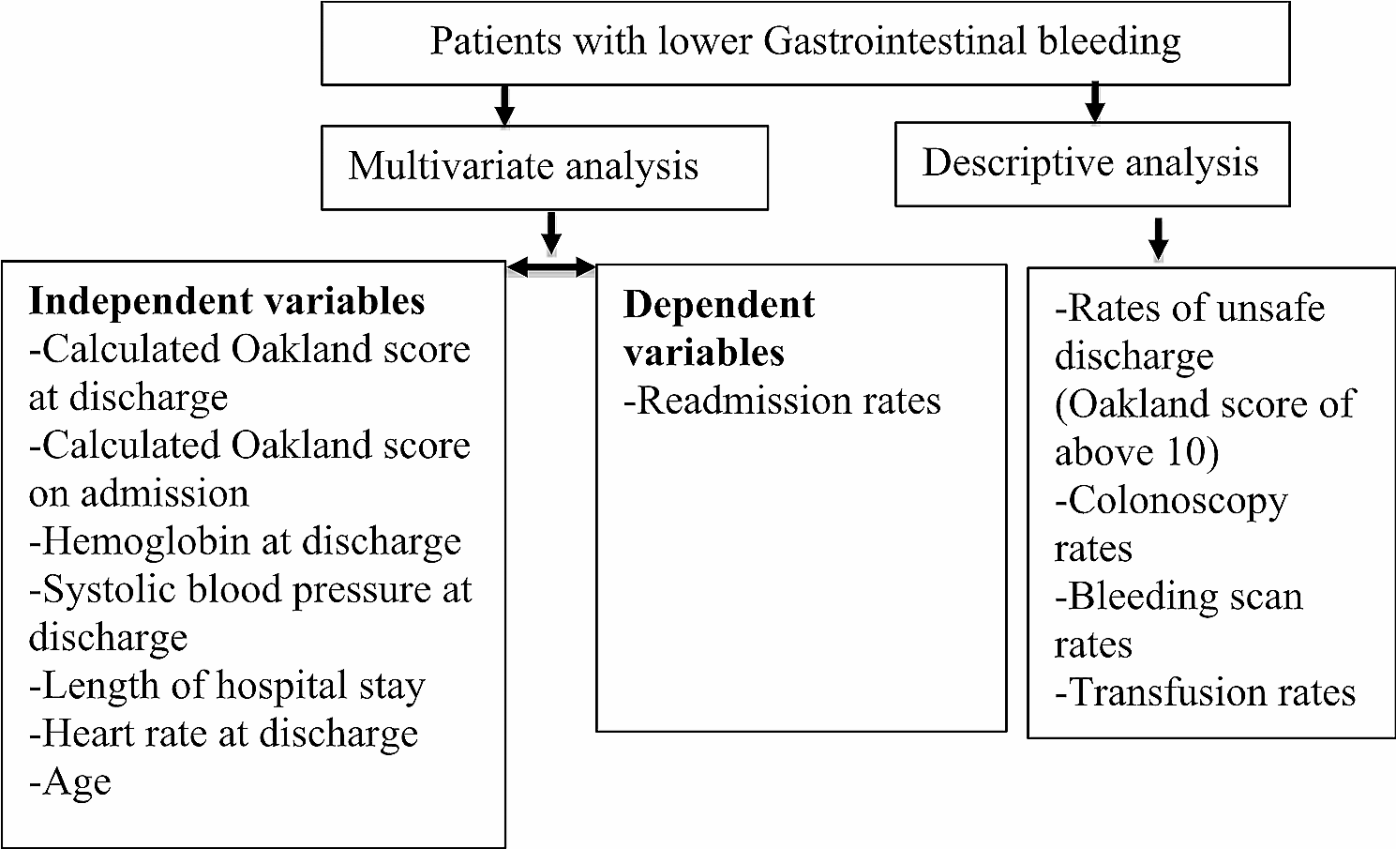
Flow chart showing the methods for statistical analysis

Statistical analysis was performed using SPSS Statistics for Windows, version 23.0 (SPSS Inc., Chicago, Ill., USA) and GraphPad Prism (Version 8.0.2, San Diego, California, USA). Probability values less than 0.05 at a 95% confidence interval (CI) were considered significant. Multivariate regression analysis was performed for the primary and secondary outcomes. Flow chart showing the methods for statistical analysis is provided in Fig. 1.

## Results

### General characteristics

A total of 108 patients were included of which 53 (49.1%) were females. The median age of participants was 65 (95% CI: 61–70) years. Among all the patients, 89 (82.4%) were African American, 17 (15.7%) Caucasian, and 2 (1.9%) others (Table 1). The median hemoglobin at baseline (prior to current hospitalization) was 10.8 g/dl; and about one third (37/108 (34.3%)) of the patients had history of previous admission for lower GI bleeding. On arrival to the hospital the median hemoglobin was 8.8 (95% CI, 8.2–9.5) g/dL; majority (69.4%) of which required transfusion resulting in an improved mean hemoglobin to 8.9 (95% CI,8.6–9.3) g/dL at the time of discharge from hospital. Most of the patients were also treated with proton pump inhibitors (80%) and underwent colonoscopy (69.4%) as shown in Table 2. Computed Tomography Angiography (CTA) use was seen in 21.3% of the cases and only 11.1% of patients had a bleeding scan. A history of anticoagulant, aspirin and clopidogrel use was present in 19.4%, 28.7%, and 8.3% of patients respectively (Table 3). Detailed description of the percentage of patients requiring more than one unit of packed red blood cells (PRBC) is provided in Table 4.

**Table 1:** Demographic characteristics of patients admitted with lower GI bleeding

Variable		Number of patients *N* = 108 (%)
Age	< 40	9 (8.3%)
	40–69	55 ( 50.9%)
	≥ 70	44 (40.7%)
Sex	Female	53 (49.1%)
	Male	55 (50.9%)
Gender	African American	89 (82.4%)
	Caucasian	17 (15.7%)
	Other	2 (1.9%)

**Table 2:** Descriptive characteristics of interventions provided to patients admitted with lower GI bleeding

Variable		Number of patients *N* = 108 (%)
Colonoscopy (Yes/No)	No	33 (30.6%)
	Yes	75 (69.4%)
CTA(Yes/No)	No	85 (78.7%)
	Yes	23 (21.3%)
Bleeding scan (Yes/No)	No	96 (88.9%)
	Yes	12 (11.1%)
PPI during hospital stay (Yes/No)	No	28 (25.9%)
	Yes	80 (74.1%)

**Table 3:** Anticoagulation and antiplatelet therapy of patients admitted with lower GI bleeding

Variable		Number of patients *N* = 108 (%)
Anticoagulation (Yes/No)	No	87 (80.6%)
	Yes	21 (19.4%)
Aspirin (Yes/No)	No	77 (71.3%)
	Yes	31 (28.7%)
Clopidogrel (Yes/No)	No	99 (91.7%)
	Yes	9 (8.3%)

**Table 4:** General outcomes of patients admitted with lower GI bleeding

Variable		Number of patients (*N*, %)
Death at discharge (Yes/No)	No	106 (98.1%)
	Yes	2 (1.9%)
AMA (Yes/No)	No	103 (95.4%)
	Yes	5 (4.6%)
One-year readmission after discharge (Yes/No)	No	99 (91.7%)
	Yes	9 (8.3%)
30-day readmission after discharge (yes/No)	No	104 (96.3%)
	Yes	4 (3.7%)
Previous LGI bleed admission	No	71 (65.7%)
	Yes	37 (34.3%)
DRE+ (Yes/No)	No	29 (26.9%)
	Yes	79 (73.1%)
Transfusion of PRBC	0 units of PRBC	42 (38.9%)
	1 unit of PRBC	17 (15.7%)
	2 units of PRBC	15 (13.9%)
	3 units of PRBC	13 (12.0%)
	4 units of PRBC	9 (8.3%)
	5 units of PRBC	6 (5.6%)
	6 + units of PRBC	6 (5.6%)
Charlson comorbidly index	Mild (0–2)	34 (31.5%)
	Moderate (3–4)	37 (34.3%)
	Severe (5 and above)	37 (34.3%)
HR at discharge	< 70	33 (30.6%)
	70–89	54 (50.0%)
	90–109	21 (19.4%)
	≥ 110	0 (0%)
SBP at discharge	≤ 89	2 (1.9%)
	90–119	27 (25.0%)
	120–129	22 (20.4%)
	130–159	45 (41.7%)
	≥ 160	12 (11.1%)
Hemoglobin at discharge	3.6–6.9	2 (1.9%)
	7.0-8.9	53 (49.1%)
	9.0-10.9	34 (31.5%)
	11.0-12.9	14 (13.0%)
	13.0-15.9	4 (3.7%)
	≥ 16.0	1 (0.9%)
Rate of poor Oakland score (> 10) at admission and discharge	Patients who had Oakland score of > 10 at admission	100 (92.6%)
	Patients who had Oakland score of > 10 at discharge	104 (96.2%)
Number of patients by baseline anemia	Baseline anemia	64 (59.3%)
	No Baseline anemia	29 (20.9%)
	No baseline anemia recorded	15 (13.9%)

On admission, 100/108 (92.6%) of patients had an Oakland score of > 10. It was notable that even more patients 104/108 (96.2%) had elevation of Oakland Score greater than 10 at discharge. Even though, the mean Oakland score slightly improved from 21.7 (95% CI, 20.4–23.1) of the day of arrival to 20.3 (95% CI, 19.4–21.2) at discharge, only 4/108 (3.7%) of patients had an Oakland score of *≤* 10 at discharge. In other words, 96.3% of patients were discharged with a high-risk Oakland score of > 10. Nonetheless, only 4/108 (3.7%) and 9/108 (8.3%) of patients required readmission during follow up at 1 month and 1 year, respectively. The mean LOS was 7.9 (95% CI, 6.6–9.1). There were two deaths among 108 patients admitted for acute lower GI bleeding. The first patient had hemorrhagic shock leading to pulseless electrical activity (PEA), while the second patient developed severe sepsis followed by PEA. (Tables 4 and 5). At the time of discharge 106/108 (98.1%) of patients had completely resolution of their active bleeding.

**Table 5:** Median and mean values for descriptive characteristics of patients admitted with lower GI bleeding

Median and mean values			
		Median (95% CI)	Mean (95% CI)
Heart rate	On day of arrival	89 (85.0–93.0)	91.4 (87.5–95.2)
	On day of discharge	78.5 (75.0–82.0)	77.0 (74.0–80.0)
SBP	On day of arrival	126.5 (120.0-130.0)	129.0 (124.0-134.1)
	On day of discharge	131 (125.0-139.0)	130.7 (125.7-135.8)
Age		65 (61–70)	64.6 (61.3–67.9)
Oakland score at discharge	Readmitted	23 (20–25)	22.7 (21.1–24.3)
	Non-readmitted	21 (20–22)	20.1(19.1–21.0)
Median and mean Oakland score in all patients	On day of arrival	24 (21–25)	21.7 (20.4–23.1)
	On day of discharge	21 (20–22)	20.3 (19.4–21.2)
Median and mean Hemoglobin (g/dl)	Baseline	10.8 (9.8–11.7)	11.0 (10.5–11.5)
	On day of arrival	8.5 (7.7–9.4)	8.8 (8.2–9.5)
	On day of discharge	8.9 (8.6–9.3)	9.4 (9.0-9.7)
Median and mean PRBC transfused	during hospital stay (in units)	1(1–2)	2.0 (1.5–2.5)
Length of stay in days	Median	6 (5–6)	7.9 (6.6–9.1)

### Multivariate analysis results

Several factors were assessed for possible association with future risk of readmission due to another lower gastrointestinal bleeding as shown in Table 6. Among all the variables, history of previous GIB was independently associated with a risk of readmission (AOR = 4.41 (95%CI: 1.010-19.348; *P* = 0.048).

**Table 6:** Factors associated with readmission among patients with lower gastrointestinal bleeding

Variable		COR	95% CI	*P* value	AOR	95% CI	*P* value
Sex	Male	1.225	0.310–4.833	0.772			
	Female	1					
Previous GI bleeding	Yes	4.387	1.030-18.694	0.046	4.421	1.010-19.348	0.048
	No	1					
Colonoscopy	Yes	0.518	0.130–2.067	0.351			
	No	1					
CTA	Yes	1.061	0.205–5.491	0.944			
	No	1					
PPI	Yes	0.676	0.157–2.904	0.598			
	No	1					
Anticoagulation	Yes	1.203	0.231–6.259	0.826			
	No						
Aspirin	Yes	2.133	0.533–8.541	0.284			
	No	1					
Hgb at discharge	< 9 g/dl	2.041	0.483–8.621	0.332			
	≥ 9 g/dl	1					
Race	AA	1.778	0.209–15.120	0.598			
	Non-AA	1					

COR: crude odds ratio; AOR: adjusted odds ratio; CTA: computed tomography angiography; PPI: Proton pump inhibitors

## Discussion

The Oakland score has been validated in the UK and US to predict safe discharge in patients who present with LGIB. However, this study demonstrated findings that question the generalizability of the Oakland score for certain patient populations. Oakland score should not be used as a sole criterion to determine discharge for patients with LGIB. In this retrospective study, at the time of discharge, almost all (96.2%) patients had a higher risk Oakland score of greater than 10, despite an overall low rate of readmission at 1-month of only 3.7% (significantly lower than national average rate of 15%). This is in large part due to baseline anemia that has the highest contribution for the Oakland score. In fact, the median Oakland score before hospitalization due to GI bleeding was at least 13 given the median hemoglobin of 10.8% at baseline. Since only 2/108 (1.8%) of patients demonstrated improved Oakland score (*≤* 10) at discharge, the use of this score does not represent a helpful tool in our patient population.

The Oakland score is a clinical predictor model which assess the safe outpatient management for patients with LGIB, it was developed and validated in United Kingdom in 2017 and externally validated in the US in 2020. At the time of validation, this score was compared with other risk scores (Blatchford, AIMS65, BLEED, Strate, NOBLADS, and Rockall) and showed a better prediction in safe discharge [2, 5]. This score model contained 7 (age, sex, previous hospitalization with LGIB, digital rectal exam, SBP, pulse and Hgb) and from those, Hgb and SBP contribute with the highest point representation, 0–22 points and 0–5 points, respectively [2, 5].

In this study of 108 patients admitted for LGIB, where the majority were African Americans, we retrospectively evaluated their admission and discharge criteria and analyzed the hypothetic use of the Oakland score to predict safe discharge. We considered the threshold point for safe discharge of less than or equal to 10, as patients with a threshold of more than 10 are believed to carry an increased adverse events risk and should be hospitalized [5]. Lower risk patients, whose bleeding had stopped can have a close follow up with a gastroenterologist in an outpatient setting [9, 10]. Spontaneous resolution of the majority of LGIB has been well documented, supporting non-hospital management of these low-risk patients [11, 12]. Assessing the main two variables of this score, the mean SBP at admission and discharge did not variate significantly, 129.0 and 130.7 respectively but represented 1-point drop in the score assessment. While the mean Hgb was 8.8 g/dL on arrival and 9.4 g/dL at discharge, also a small improvement but represented 4 points drop in the score assessment. During the original validation of Oakland score, a mean of 9.7 g/dL was found in the not safely discharge group compared with 12.9 g/dL in the safely discharged group, this represented a moderate improvement in Hgb, reflecting 5 points drop in the score assessment [2]. The external validation of this score in the US, a mean of 8.5 g/dL was found in the not safely discharge group compared with 12.2 g/dL in the safely discharged group, this represented a considerable improvement in Hgb, reflecting 9 points drop in the score assessment [5]. With this important impact of Hgb in the Oakland score, we evaluated baseline Hgb in our population and nearly 60% of the patients had history of anemia which we believe might have contribute to the differences seen in our study.

Considering our findings, the use of the Oakland score was not a helpful tool for safe discharge in African American patients who present with LGIB. Comparing our discharged group with Oakland score greater than 10 with the same group in the original development cohort [2], similar mortality and readmission rate was found, 1.9% and 3.7% vs. 2% and 5%, respectively. Nevertheless, if we take in consideration patient baseline anemia, a criteria not contemplated in the original study, more than 30% of the patients that were admitted with Oakland score > 10 and did not require blood transfusion could have been followed in the outpatient setting, avoiding an unnecessary hospital admission. Assessing readmission, we found that history of admission for previous LGIB was associated with readmission (*p* = 0.046). Similar findings have been found in 30-day readmission rate assessment in a prospective observational study of a single center and retrospective evaluation of a national sample, showing a high readmission rate due to recurrent LGIB [13, 14]. Another study, using the United States National Readmission Database, showed that LOS, associated comorbidities, and GI diseases were the major predictors of readmission in 30 days, with higher rate (14.6%) of another episode of GI hemorrhage among all GI diseases [15]. In the secondary analysis of our study, important findings as PPI use in LGIB was assessed. The inappropriate use of PPI in hospitalized patients has been of great research interest due increase risk of infections and costs [16, 17]. Data from PPI use in the setting of LGIB is scarce, a retrospective study showed that more than 30% of patients admitted for LGIB were on PPI, around of 46% of those patients received this medication without indication [18]. In our study, 74% of patients received PPI during hospital stay. An important finding to increase awareness among clinicians of appropriate use of PPI as a systematic review and meta-analysis showed an increased risk of small bowel damage using this medication in patients taking nonsteroidal anti-inflammatory drug (NSAID) [19].

There are several limitations to our study. First, this was a single center retrospective study, the sample size was small, and the retrospective description of this data can only determine association but not causality. Second, patient inclusion criteria were based on ICD-10 codes and some patients can potentially be included as LGIB, but may have had small-bowel bleeding. Third, nearly 30% of patients did not undergo colonoscopy during hospitalization, making some final diagnoses not completely reliable. Fourth, around 14% of patient’s baseline Hgb data was not available. This missing data could increase the percentage of patients with previously diagnosed anemia. Lastly, patients can have readmission to an outside hospital which would not have been captured in our readmission rates.

## Conclusion

In this single center study, nearly all patients who had Oakland score of > 10 at admission continued had persistent elevation at the time of discharge. If the Oakland Score was used as the sole criteria for discharge in our population, the majority of patients would not have met discharge criteria. However, despite this the majority of these patients did not require readmission for LGIB. In our population the Oakland score did predict safety of discharge. We believe this variation from prior cohorts is due to the presence of baseline anemia in our population. Further prospective studies considering baseline anemia would be helpful to further evaluate the validity of the Oakland score in this patient population.

## Data Availability

The data information used and/or analyzed during this study are available from the corresponding author (LMN) on reasonable request.

## Abbreviations

LGIB: Lower gastrointestinal bleeding
WAMC: WellStar Atlanta Medical Center
SBP: Systolic blood pressure
Hgb: Hemoglobin
GIB: Gastrointestinal bleeding
EHR: Electronic health record
CCI: Charlson Comorbidity index
ICU: Intensive care unit
LOS: Length of stay
CI: Confidence Interval
CTA: Computed Tomography Angiography
PRBC: Packed red blood cells
PEA: Pulseless electrical activity
